# Evasion of vaccine-induced humoral immunity by emerging sub-variants of SARS-CoV-2

**DOI:** 10.2217/fmb-2022-0025

**Published:** 2022-03-30

**Authors:** Takahiko Koyama, Kei Miyakawa, Reitaro Tokumasu, Sundararaj S Jeremiah, Michiharu Kudo, Akihide Ryo

**Affiliations:** ^1^IBM Thomas J. Watson Research Center, Yorktown Heights, NY 10583, USA; ^2^Department of Microbiology, Yokohama City University, Yokohama, Kanagawa, Japan; ^3^IBM Research – Tokyo, Tokyo, Japan

**Keywords:** Beta variant, COVID-19, immune escape, Mu variant, SARS-CoV-2, vaccine effectiveness

## Abstract

**Background:** Emergence of vaccine-escaping SARS-CoV-2 variants is a serious problem for global public health. The currently rampant Omicron has been shown to possess remarkable vaccine escape; however, the selection pressure exerted by vaccines might pave the way for other escape mutants in the near future. **Materials & methods:** For detection of neutralizing antibodies, the authors used the recently developed HiBiT-based virus-like particle neutralization test system. Sera after vaccination (two doses of Pfizer/BioNTech mRNA vaccine) were used to evaluate the neutralizing activity against various strains of SARS-CoV-2. **Results:** Beta+R346K, which was identified in the Philippines in August 2021, exhibited the highest vaccine resistance among the tested mutants. Surprisingly, Mu+K417N mutant exhibited almost no decrease in neutralization. Imdevimab retained efficacy against these strains. **Conclusions:** Mutations outside the receptor-binding domain contributed to vaccine escape. Both genomic surveillance and phenotypic analysis synergistically accelerate identifications of vaccine-escaping strains.

## Plain language summary

Prior to the Omicron variant, the SARS-CoV-2 Beta sub-variant found in the Philippines in August 2021 exhibited remarkable vaccine-escaping capacity. Although Omicron is, at the time of writing, causing most of the infections globally, both genomic surveillance and phenotypic analysis should be reinforced to accelerate the identification of newly emerging vaccine-escaping SARS-CoV-2 variants.

## Tweetable abstract

The Omicron variant and the Beta sub-variant harboring a spike-R346K mutation possess a high potential to escape vaccine immunity.

SARS-CoV-2, the causative agent of COVID-19, has been rapidly evolving over time ever since its outbreak in Wuhan, China, in late 2019. Despite the proofreading function of exonuclease, the virus evolves at the rate of 1.12 × 10^-3^ mutations per site per year [1]. One of the salient mutations, spike-D614G, appeared in January 2020 and soon became ubiquitously dominant by April [2]. As vaccine development was initiated almost immediately after the pandemic started, current major vaccines, including BNT162b2 [3], mRNA1273 [4] and ChAdOx1-S [5], are all based on the original strain without D614G. In late September, the Alpha variant (B.1.1.7) harboring the N501Y mutation that conferred higher infectivity emerged in the UK [6] and spread rapidly all over the world before vaccines became widely available in December. Fortunately, despite those mutations, vaccines have demonstrated an effectiveness against most existing strains prior to the Omicron variant (BA.1) of late 2021 [7,8]. Interestingly, the Beta variant (B.1.351), first identified in South Africa in late 2020, was found to possess a higher immunity escape capacity. Despite this evolutionary advantage, the Beta variant did not spread much, as compared with the Alpha variant. In April 2021, another new strain, the Delta variant (B.1.617.2), emerged in India [9] and spread even more rapidly than the Alpha variant. The Delta variant has higher infectivity than the Alpha [10], and breakthrough infections were reported in numerous places, including the USA [11] and Israel [12], where the majority of the population was fully vaccinated by the summer of 2021, as shown in Figure 1 [13]. Studies suggest that vaccines are still effective against the Delta variant and its sub-strains [14], and the breakthrough infections principally occurred due to waning vaccine effectiveness in people who had received early vaccinations or in the elderly [15]. In June, another variant harboring E484K and R346K was found in Angola with low neutralization titer against mRNA1273 vaccinated serum [16]. In late November 2021, the emergence of the Omicron variant (B.1.1.529/BA.1) with 32 mutations in the spike protein, in South Africa, shook the world, and Omicron spread with unprecedented speed, even faster than the Delta variant. As fully vaccinated people were also infected, the Omicron variant was considered to possess high immunity escape capacity according to the epidemiological data [17] and its immunity escape capacity was confirmed experimentally [8]. To answer the question of whether immunity escape against existing vaccines or prior infections is prevalent, the authors have conducted a neutralizing antibody study against a fictitious strain, the Beta variant with spike-R346K mutation (Beta+R346K). Surprisingly, the strain has recently emerged in the Philippines (EPI_ISL_5556206) [18,19]. Similarly, the Mu variant (B.1.621) with spike-K417N mutation (Mu+K417N) has been identified not only in the South American countries, such as Columbia and Peru, but also in North America and European nations. Since it was observed in October in Peru (EPI_ISL_6208403, EPI_ISL_5645353, EPI_ISL_5645484), Spain (EPI_ISL_6026625), Belgium (EPI_ISL_5779882) and the USA (EPI_ISL_5522122), the strain might still be active. Beta+R346K and Mu+K417N share the same haplotype with R346K, K417N, E484K and N501Y mutations in the receptor-binding domain (RBD) in the spike protein, as shown in Figure 2. In this study, the authors investigated neutralization antibody titer against these strains using sera from individuals in Yokohama, Japan, after receiving the second dose of the BNT162b2 vaccine. Around the time of the study, the vaccination rate was rapidly increasing in Japan, as seen in Figure 1, and the Delta variant had started to retreat.

**Figure 1.**
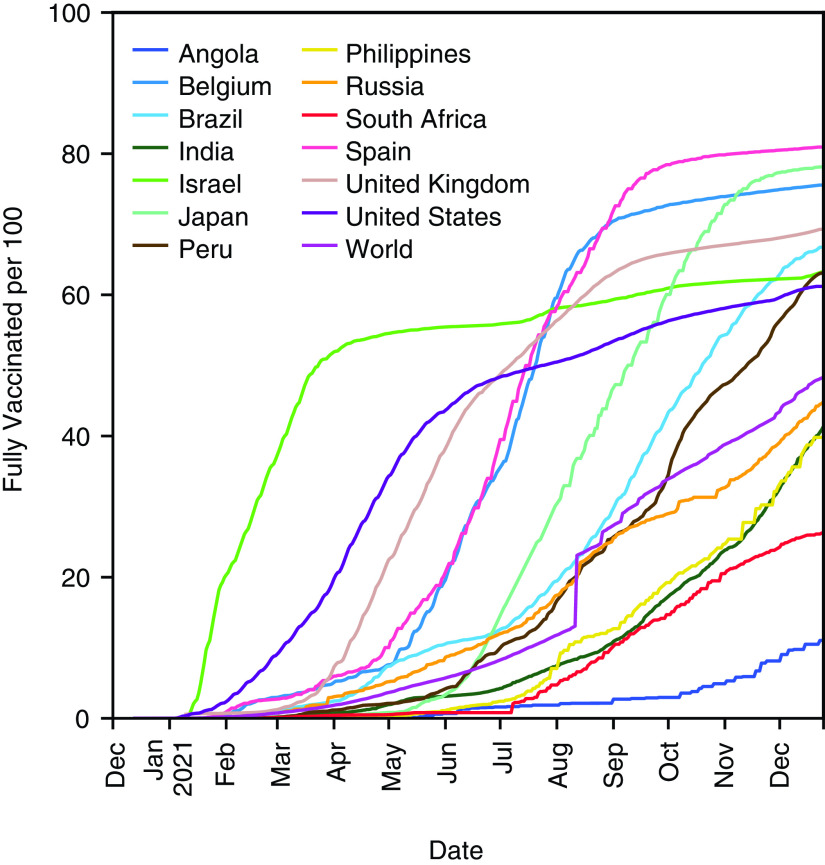
Full vaccination status per 100 of the various countries and the world average. The graph is based on *Our World in Data* taken from [13].

**Figure 2.**
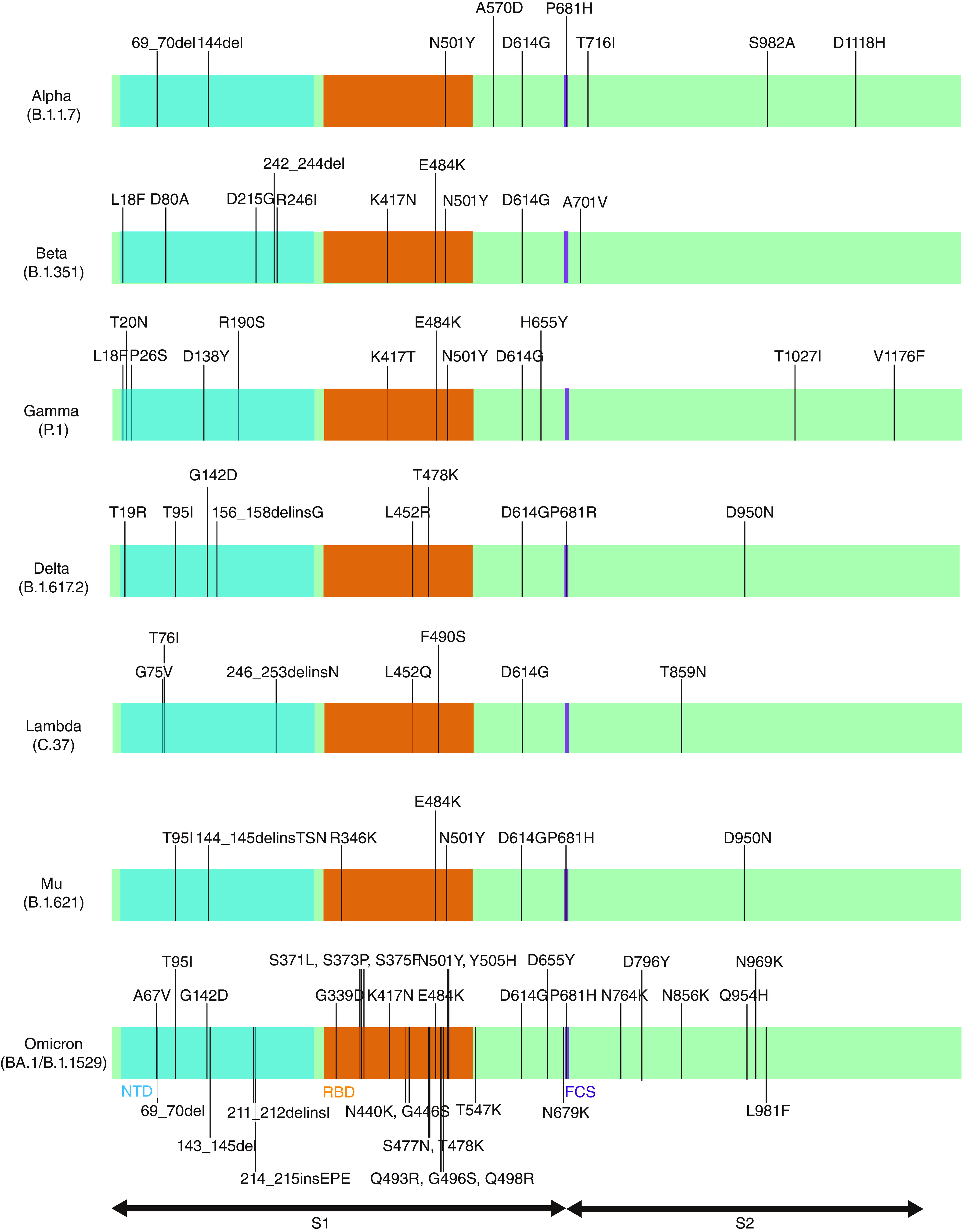
Spike protein mutations in the Alpha, Beta, Gamma, Delta, Lambda, Mu and Omicron variants. The Beta does not harbor R346K, whereas the Mu lacks K417N in the receptor-binding domain. FCS: Furin cleavage site; NTD: N-terminal domain; RBD: receptor-binding domain.

## Material & methods

### Subjects & ethics statement

Participants were recruited from among the medical staff of Yokohama City University Hospital in March 2021. Written informed consent was obtained from all the participants. Blood samples were collected 1 week after administration of the second dose of Pfizer/BioNTech mRNA vaccine. The authors randomly selected a set of 19 samples with blinding to demographic characteristics. All samples were tested for antibodies against SARS-CoV-2 nucleocapsid protein (NP) and were confirmed to be negative (Table 1). This study was approved by the Yokohama City University Certified Institutional Review Board (reference no. B210300001), and the protocols used in the study were approved by the ethics committee.

**Table 1.** Summary of BNT162b2-vaccinated sera used in this study.

ID	Age	Sex	NP-IgG
1	57	Female	Negative
2	54	Female	Negative
3	54	Female	Negative
4	50	Female	Negative
5	51	Female	Negative
6	53	Male	Negative
7	49	Female	Negative
8	53	Female	Negative
9	48	Female	Negative
10	48	Female	Negative
11	48	Female	Negative
12	47	Female	Negative
13	52	Female	Negative
14	46	Female	Negative
15	45	Female	Negative
16	62	Male	Negative
17	44	Female	Negative
18	45	Female	Negative
19	43	Female	Negative

### Calculation of median neutralizing titer

The authors performed the rapid neutralization test (HiBiT-based virus-like particle neutralization test [hiVNT]) for calculating neutralizing titer, as described previously [20]. Briefly, the target cells (Vero E6/TMPRSS2-LgBiT cells) seeded in 96-well plates were inoculated with 50 μl of HiBiT-tagged virus-like particles (hiVLPs) containing diluted serum (1:20–1:43,740 dilution). Intracellular luciferase activity was measured 3 h after inoculation.

### Calculation of median effect concentration

Vero E6/TMPRSS2-LgBiT cells were inoculated with 50 μl of hiVLPs containing diluted antibody (final concentration of 0.64–50,000 ng/ml). The concentration of the antibody that resulted in a 50% reduction in luminescence compared with that of the non-antibody control was determined as EC_50_.

## Results

The authors recently developed the hiVNT system to detect neutralizing antibodies in a low-biosafety setting [20]. This is a simple, high-throughput assay system for the rapid determination of SARS-CoV-2 neutralizing antibodies in the sera of individuals. Serum dilutions showing 50% inhibition of infection (NT_50_) were determined for the Alpha B.1.1.7 (Alpha), B.1.351 (Beta), P.1 (Gamma), B.1.617.2 (Delta) and B.1.1.529 (Omicron) lineages of SARS-CoV-2 as variants of concern (VOCs), lineages C.37 (Lambda) and B.1.621 (Mu) as variants of interest (VOIs) and further derivatives derived from Beta, Delta and Mu strains.

**Figure 3.**
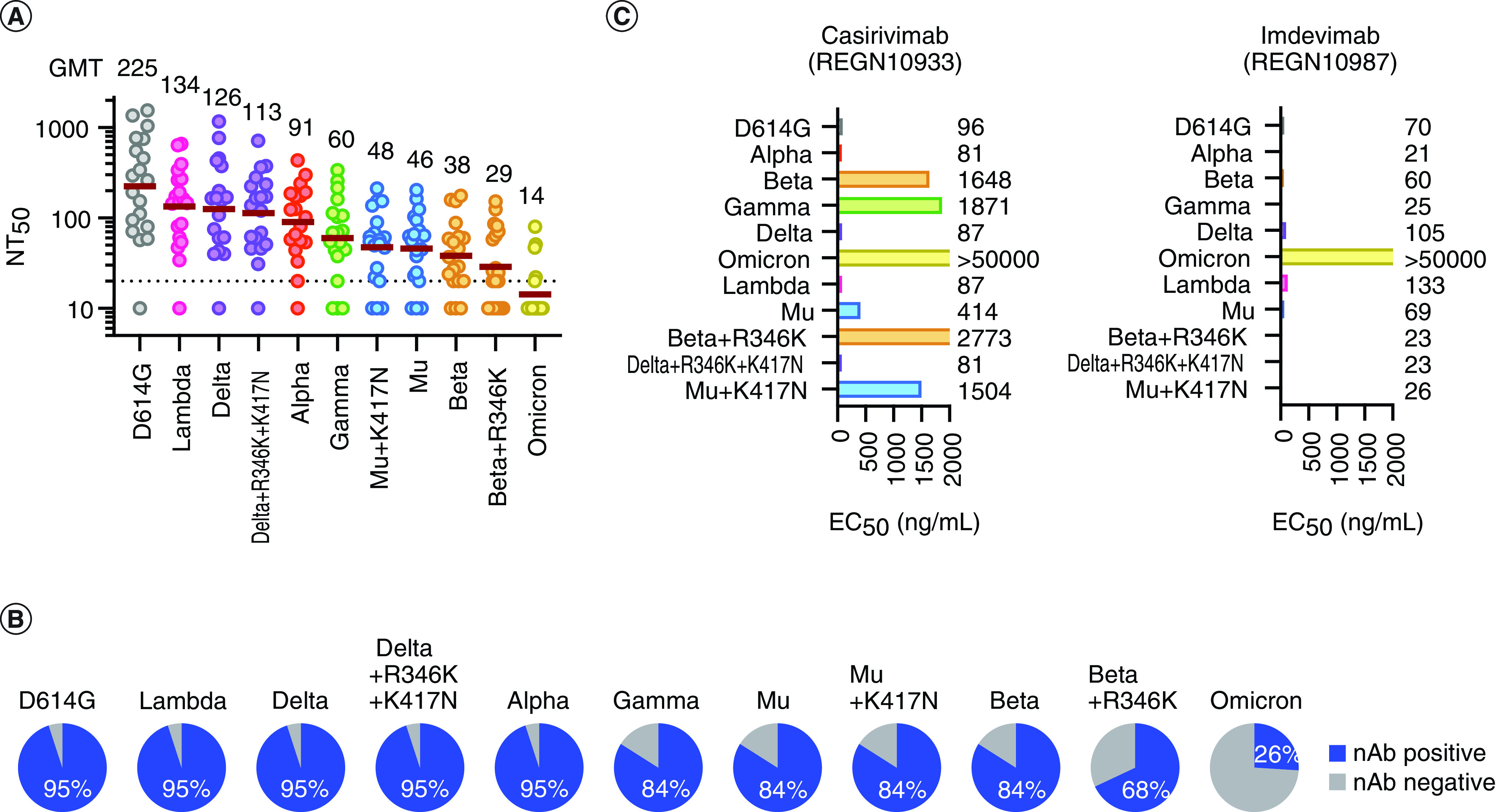
Immune-escape features of Beta+R346K and Omicron variants. **(A)** Neutralization titers of variant of concerns, variants of Interest, Beta+R346K, Delta+R346K+K417N and Mu+K417N. The mean of two independent determinations is plotted. The brown lines indicate the geometric mean titers, the values of which are displayed above the graph. **(B)** Rate of vaccinees' sera positive for neutralizing antibodies against indicated variants. **(C)** Neutralization of each mutant strain by a dual-antibody cocktail. The numbers indicate the EC_50_, ng/ml, determined by two independent experiments.

The NT_50_ of the Beta+R346K strain was found to be substantially lower than any strains found prior to Omicron variant, as shown in Figure 3A. The Beta, the Delta with spike protein mutations R346K and K417N (Delta+R346K+K417N) and the Mu were considered partially immunity-escaping with NT_50_ values of 38, 113 and 46, respectively. The NT_50_ value of the Beta+R346K was much lower, with a value of 29, indicating that infection with this variant can occur even when vaccination effectiveness is at its prime. The contribution of R346K mutation was more apparent in the positive rate of neutralizing antibodies in vaccinated sera, as shown in Figure 3B. While R346K mutation barely contributed to the Mu or the Delta variant, a notable difference was observed for the Beta variant.

As vaccination effectiveness wanes over time, the chances of breakthrough infections tend to increase. This can cause huge numbers of vaccinated yet susceptible individuals in a population, leading to a surge of new cases that may potentially collapse medical systems. Since Beta+R346K and Mu+K417N share the same haplotype within the RBD of the spike protein, the authors expected that the neutralization antibody titer would substantially drop for Mu+K417N. On the contrary, no substantial decrease in neutralization antibody titer was observed for Mu+K417N as compared with the original Mu strain. Thus, the mutational differences in other domains must have contributed to the reduced neutralization titer. The Beta variant has more mutations than the Mu variant in the N-terminal domain (NTD), as shown in Figure 2. Furthermore, none of the additional mutations K417N and R346K to the Delta variant altered its NT_50_ values.

Likewise, the efficacy of the Regeneron cocktail, which consists of two monoclonal antibodies, casirivimab and imdevimab, was evaluated for these strains. Besides the Omicron variant; the Beta strain, Beta+R346K, and the Gamma strain exhibited resistance to casirivimab; however, imdevimab retained efficacy to these strains, excluding the Omicron variant, as shown in Figure 3C.

## Discussion

As of November 2021, only 50% of the world's population are fully vaccinated [13]. According to the recent study by Tartof *et al.*, the protective efficacy of vaccines against infection declines rapidly from over 90% to below 50% within 6 months in almost all age groups [21]. Similar observations of waning vaccine effectiveness have been reported by Goldberg *et al.* [12] and others. Choi *et al.* reported that neutralization titer against the Beta variant, which harbors K417N and E484K, declined substantially compared with the Alpha variant [16]. In the same paper, they identified reduced neutralization titer for A.VOI.V2 found in Angola. A.VOI.V2 harbors R346K, T478R and E484K in the RBD. Also, Miyakawa *et al.* [22] as well as Uriu *et al.* [23] reported that neutralization efficacy was reduced for the Mu variant harboring R346K, E484K and N501Y in the RBD. These neutralization studies led the authors to hypothesize that strains with R346K, K417N and E484K might induce vaccine immune escape.

It was quite surprising to observe a prominent difference in neutralization titer between Beta+R346K and Mu+K417N, despite their being the same RBD haplotype. The study conducted by Liu *et al.* suggests that neutralizing and infection-enhancing antibodies against the NTD plays a role in infectivity [24]. The two strains are quite different in the NTD. Whereas Mu is simple, with one single-nucleotide variant (SNV), T95I, and a deletion, 144_145delinsTSN, Beta harbors three SNVs – D80A, D215G and R246I – and a deletion, 242_244del, as illustrated in Figure 1. Another question is why Beta+R346K did not proliferate despite its immune-escaping capacity. Kepler *et al.* decomposed the fitness of pathogens into intrinsic factors such as viral genetics and extrinsic factors such as climate and host factors [25]. Unlike the Alpha and the Delta variants, proliferation of the Beta variant was quite limited. Thus, it appears that the Beta did not have superior fitness factors with respect to the competing variants.

Since the speed of development and distribution of vaccines take time, any potential strain harboring these three mutations, among others, must be monitored [26–28]. There are still regions with low surveillance rates and sluggish reporting of SARS-CoV-2 genomes on Global Initiative on Sharing All Influenza Data (GISAID) [18,19]. It is important to eliminate such blind spots to quickly identify emerging strains with different clinical characteristics. At the same time, reverse zoonosis events have been reported in wild animals, such as deer [29], as well as in farm animals [30]. It is known that viruses evolve differently in species and might have completely different disease profiles. Therefore, it might be necessary to expand the surveillance to other species.

## Limitation of this study

This study only includes sera from donors fully vaccinated with BNT162b2. Results might differ with other vaccines such as mRNA-1273 and ChAdOx1-S. In particular, mRNA-1273 might provide better protection against the Beta+R346K strain, since mRNA-1273 seems more effective than BNT162b2 in general [31].

## Conclusion

This study identified Beta+R346K as exhibiting the highest vaccine-escaping capacity among the strains prior to the Omicron variant. On the other hand, the neutralization titer against Mu+K417N, which shares the same haplotype in the spike RBD, did not changed. Although the RBD in the spike protein is crucial in binding to ACE2 of host cells, the neutralization titer is not solely determined by mutations in the RBD. It is essential to conduct genomic surveillance of SARS-CoV-2 and phenotype screenings to preemptively minimize the threat of variants.

## Future perspective

More comprehensive genomic surveillance will be achieved. Ideally, all samples from human cases will be sequenced and surveillance will be extended to animals as well. An *In vitro* assay for the neutralization antibody titer such as hiVNT will be conducted one day.

**Summary points**

- Beta sub-variant with spike-R346K mutation had remarkable vaccine-escaping capacity prior to Omicron.
- Beta+R346K was identified in the Philippines in August 2021.
- Unexpectedly, the Mu sub-variant with spike-K417N, which has a receptor-binding domain mutation identical to that of Beta+R346K, did not exhibit more escaping capability than the regular Mu variant.
- Imdevimab exhibited efficacy against both Beta+R346K and Mu+K417N.
- Mutations in N-terminal domain play a remarkable role in vaccine escape as well as mutations in the receptor-binding domain, where the most neutralization antibodies are believed to be bound.
- The Omicron variant is not the only variant with immune-escaping capacity.
- It is imperative to eliminate blind spots in SARS-CoV-2 genomic surveillance.
- Comprehensive genomic surveillance and phenotypic screening are critical to characterize and identify variants of clinical significance in a timely manner.
